# Parasympathetic activity correlates with subjective and brain responses to rectal distension in healthy subjects but not in non-constipated patients with irritable bowel syndrome

**DOI:** 10.1038/s41598-019-43455-5

**Published:** 2019-05-14

**Authors:** Michiko Kano, Makoto Yoshizawa, Keiji Kono, Tomohiko Muratsubaki, Joe Morishita, Lukas Van Oudenhove, Mao Yagihashi, Shunji Mugikura, Patrick Dupont, Kei Takase, Motoyori Kanazawa, Shin Fukudo

**Affiliations:** 1 Sukawa Clinic, Kirari Health Coop, Fukushima, Japan; 2 Behavioral Medicine, Graduate School of Medicine, Tohoku University, Sendai, Japan; 3 Research Division on Advanced Information Technology, Cyberscience Center, Tohoku University, Sendai, Japan; 4 Laboratory for Brain-Gut Axis Studies (LaBGAS), Translational Research Center for Gastrointestinal Disorders (TARGID), KU Leuven, Leuven, Belgium; 5 Dept. Biodesign, Clinical Research, Innovation and Education Center (CRIETO), Tohoku University Hospital, Sendai, Japan; 6 Diagnostic Radiology, Tohoku University Hospital, Sendai, Japan; 7 Laboratory for Cognitive Neurology, KU Leuven, Leuven, Belgium

**Keywords:** Sensory processing, Irritable bowel syndrome

## Abstract

The nociceptive and autonomic nervous systems (ANS) are significantly intertwined. Decoupling of these systems may occur in pathological pain conditions, including irritable bowel syndrome (IBS). We investigated ANS activity and its association with visceral perception and brain activity during rectal distention in 27 patients with non-constipated IBS and 33 controls by assessing heart rate variability (HRV) using electrocardiography at rest, before, and during colorectal distention. Brain responses to colorectal distention were measured using functional magnetic resonance imaging and correlated with individual ANS function parameters. The IBS group displayed blunted sympathovagal balance [low/high-frequency ratio (LF:HF) of HRV] in response to colorectal distention compared with controls (*P* = 0.003). In controls, basal parasympathetic tone (HF component of HRV) was significantly negatively correlated with toleration threshold to the rectal distention, but not in patients with IBS (group comparison *P* = 0.04). Further, a positive correlation between baseline HF values and neural responses to rectal distension was found in the right caudate, bilateral dorsolateral anterior cingulate cortex, and pregenual anterior cingulate cortex in the control group but not in the IBS group. The results indicate abnormal interactions between ANS activity and the brain mechanisms underlying visceral perception in patients with IBS.

## Introduction

The autonomic nervous system (ANS) is the principal neural interface through which the brain and viscera interact^1^, allowing the brain to process information from the periphery and regulate visceral activity, thereby maintaining homeostasis^1^. Malfunctioning of this system can lead to a variety of physical symptoms, such as light-headiness, palpitations, diarrhea, fatigue, and pain^2^ and may comprise the core pathology of functional somatic syndromes (FSS)^3,4^. Pain is one of the main symptoms of FSS, such as irritable bowel syndrome (IBS) and fibromyalgia^3,4^, which feature amplified pain sensitivity and autonomic dysfunction^5,6^. Brain systems regulating ANS function are closely coupled with systems modulating pain processing and perception, highlighting the importance of functional interactions of these systems in pain regulatory processes and pain disorders^7^. However, the characterization of these interactions in living humans has been unsatisfactory in previous studies.

IBS is characterized by chronic recurring abdominal pain and altered bowel habits (e.g., diarrhea and constipation) in the absence of detectable organic causes with routine clinical examinations^8^. IBS is a multifactorial disorder with a complex biopsychosocial pathophysiology. The pathophysiological mechanisms involved in IBS may vary between subjects, but include altered gastrointestinal motility^9^, visceral hyperalgesia^9^, increased intestinal permeability^10^, immune activation^10^, altered microbiota^11^, and disrupted communication between the gastrointestinal tract and the central nervous system^12^. This has led to the concept of IBS as a disorder of brain–gut interaction^10,13^. The sympathetic and parasympathetic branches of the ANS mediate the bidirectional brain-gut communication largely by modulating the third ANS branch, the enteric nervous system^5^.

The ANS can impact on gastrointestinal functions, such as permeability^10^, immune function^10^, and gut flora^14^. These effects may induce higher peripheral sensitivity to pain by modifying visceral afferent signals^10^. These signals are conveyed to the nucleus of the solitary tract (NTS) and then to the parabrachial nucleus (PBN) in the brainstem through afferent vagal and spinal pathways that stimulate reflex arcs, thereby engaging autonomic responses. The PBN is the main integrated site for all afferent homeostatic signals, which in turn projects to forebrain regions, including the thalamus, the anterior cingulate cortex (ACC), the insula, hypothalamus, and amygdala^10,15,16^. Inputs from the PBN and these forebrain regions are received by the rostral ventromedial medulla (RVM) and periaqueductal grey (PAG), central autonomic effector regions^10,16,17^. These top-down effects modulate spinal afferent signal transmission at the dorsal horn level through descending modulatory projections, and control sympathetic and parasympathetic ANS outputs^10,15^. A neuroimaging study demonstrated that these descending pain modulatory circuits in the brainstem are dysregulated in patients with IBS^18^. Together with the autonomic dysfunction that characterizes IBS, disruption of brain-mediated ANS function and pain processing is suggested.

The central autonomic control areas form the central autonomic network (CAN). This network largely overlaps with the network involved in pain perception; including a strong integration at the level of the dorsal horn, the brainstem, and the thalamus, hypothalamus, amygdala, insular cortex, and ACC in the forebrain^19^. Studies demonstrated that measures of pain perception are associated with autonomic functions such as heart rate variability (HRV)^7^. Greater parasympathetic activity is related to lower pain perception in healthy subjects^20–22^, patients with fibromyalgia^23^, and those with chemotherapy-induced neuropathy pain^24^. The anatomical correspondence between CAN and pain processing networks implies that the brain may mediate this close association between the ANS and pain perception.

Although empirical studies suggest a close interaction between autonomic function and pain processing and modulation, research on the association of these functions in patients with pathological pain conditions remains scarce. Thus, we aimed to investigate the relation between autonomic function and visceral pain perception, as well as brain responses to visceral pain in a healthy state and in the context of functional abdominal pain (i.e., IBS). We assessed first whether sympathetic and parasympathetic autonomic reactivity to visceral stimulation differs between patients with non-constipated IBS and controls. We also examined whether individual differences in autonomic activity correlate with the perception of or brain responses to colorectal distention. Finally, we evaluated whether these correlations between autonomic activity and perceptual and brain responses vary between controls and patients with non-constipated IBS.

## Results

### Subject characteristics

This study enrolled 27 patients with IBS (12 males; mean age: 22 ± 2.8 years) diagnosed according to the ROME III criteria^25^ and age and sex matched 33 healthy controls (16 males; mean age: 22.3 ± 2.8 years). All patients belonged to the non-constipated subtype (24 diarrhea-predominant subtype [IBS-D] and 3 mixed subtype [IBS-M]). The exclusion criteria were a history of any mental and organic diseases including abdominal surgery or any cardiovascular or respiratory diseases. The average score ± standard deviation (SD) for the Self-Rating Depression Scale (SDS)^26^, the State–Trait Anxiety Inventory (STAI)^27^ trait score, and IBS severity Index (Symptom Severity Scale, IBS-SI)^28^ were 36.0 ± 6.4, 36.4 ± 6.08, and 53.5 ± 47.2, respectively for controls, and 38.4 ± 8.9, 42.4 ± 12.3, and 177.0 ± 52.4 respectively for patients with non-constipated IBS (Table 1). Patients with IBS exhibited significantly higher scores on the IBS Severity Index (*P*_(*Holm*)_ < 0.0001), but not anxiety trait (*P*_(*Holm*)_ = 0.06) and depressive score (*P*_(*Holm*)_ = 0.32) compared with healthy controls (HCs) with stepdown Bonferroni (Holm) correction for multiple comparisons.

**Table 1:** Subject characteristics.

(mean ± SD)	Controls (n = 33, 16 males)	non-constipated IBS (n = 27, 12 males)	*p*
age	22.3 ± 2.8	22.0 ± 2.8	
**Questionnaires**			
SDS	36.0 ± 6.4	38.4 ± 8.9	0.32
STAI (trait)	36.4 ± 6.08	42.4 ± 12.3	0.06
IBSSI	53.5 ± 47.2	177.0 ± 52.4	<0.0001

Abbreviations: IBS, irritable bowel syndrome, IBSSI, IBS severity index, SDS, the Self-Rating Depression Scale, STAI, State–Trait Anxiety Inventory.

### Perception of colorectal distention

The perception threshold of colorectal distention was assessed with the ascending method of limits (AML), in which bag pressure at rectum was increased gradually. We determined the toleration threshold, i.e., the pressure intensity at which a subject could not tolerate an increase in stimulus during a tonic stimulation lasting 3 min for measuring autonomic responses. The toleration thresholds in the control and non-constipated IBS groups were 25.9 ± 7.3 and 22.1 ± 4.1 mmHg, respectively (*P* = 0.02; Table 2). Due to the individual titration, subjective ratings of pain, urgency, and discomfort after tonic distention, were not statistically different (with stepdown Bonferroni (Holm) for multiple comparisons) between the control and non-constipated IBS groups (Table 2).

**Table 2:** Perception of colorectal distention.

(mean ± SD)	Controls (n = 33)	non-constipated IBS (n = 27)	*P*
**Toleration threshold**			
(mmHg)	25.9 ± 7.3	22.1 ± 4.1	0.02
**Rating at tonic distention**			
Pain	3.35 ± 3.04	4.76 ± 2.89	0.33
Urge	7.8 ± 1.4	7.4 ± 1.44	0.58
Discomfort	6.07 ± 2.35	6.52 ± 1.92	0.58

Abbreviations: IBS, irritable bowel syndrome.

### Autonomic responses to colorectal distention between groups

We estimated ANS activity from electrocardiogram (ECG) during a 5 min baseline (before insertion of the distention bag in the rectum), a 3 min resting period with the bag in the rectum, and a 3 min tonic distention at the individually titrated toleration threshold of each subject. The high frequency (HF; 0.15–0.40 Hz) and low frequency (LF; 0.04–0.15 Hz) bands of the heart rate variability (HRV) power spectrum were evaluated. We used the HF band as a maker of the parasympathetic activity and the LF:HF ratio as an indicator of the sympathovagal balance^29^. After ECG data quality control, the data of 25 patients with non-constipated IBS and 31 controls could be used for the final HRV analysis. The values for LF, HF and heart rate (HR) during the three periods are displayed in Table 3. As anxiety has been shown to influence HRV^30^, the correlation between the trait STAI score and the values of LF:HF and HF were performed. Pearson’s correlation between STAI trait score and baseline HF (r = 0.02, p = 0.98), LF:HF (r = 0.01, p = 0.94), ΔHF (r = 0.01, p = 0.97), and Δ(LF:HF)(r = −0.001, p = 0.99) were not significant when using all subjects. Therefore, the influence of STAI score was not taken into account in the analysis of autonomic responses to colorectal distention between groups.

**Table 3:** Values of autonomic parameters.

(mean ± SD)	Controls (n = 31)			non-constipated IBS (n = 25)		
	Baseline	Rest	Distention	Baseline	Rest	Distention
Ln (HF)	2.32 ± 0.36	3.2 ± 0.71	3.08 ± 0.62	2.25 ± 0.26	3.36 ± 0.62	3.16 ± 0.62
Ln (LF/HF)	−0.05 ± 0.3	0.05 ± 0.5	0.25 ± 0.5	−0.01 ± 0.2	0.02 ± 0.44	0.09 ± 0.38
Ln (HR)	4.13 ± 0.18	4.13 ± 0.18	4.22 ± 0.17	4.18 ± 0.14	4.13 ± 0.12	4.20 ± 0.16

Abbreviation: IBS, irritable bowel syndrome; HF, high frequency; HR, heart rate; LF, low frequency; Ln, natural logarithm.

#### Sympathovagal balance (LF:HF)

**Figure 1:**
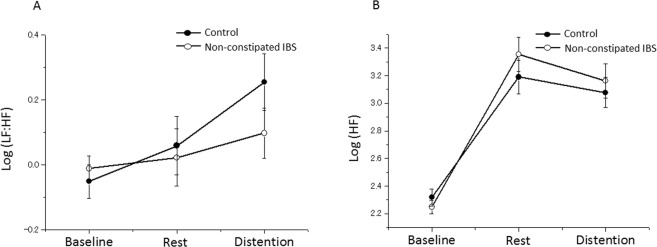
Autonomic responses to colorectal distention. (**A**) Sympathetic vagal balance (LF:HF). (**B**) Parasympathetic vagal tone (HF). Error bars represent the standard error on the mean. HF, high frequency; IBS, irritable bowel syndrome; LF, low frequency; PreDist, pre-distention.

The LF:HF ratio exhibited significant differences between conditions (i.e., baseline,, resting after the barostat bag placement, and colorectal distention; main effect of condition, *F*_(2, 111)_ = 9.05; *P* = 0.0002). While a group × condition interaction (*F*_(2, 111)_ = 6.28; *P* = 0.003) was reported, no main effects of group (*F*_(1, 57)_ = 1.03; *P* = 0.31) and sex (*F*_(1, 57)_ = 1.22; *P* = 0.27) were observed (Fig. 1A). The within-group post-hoc analysis revealed that the LF:HF ratio was significantly different between the conditions in the control group (*F*_(2, 64)_ = 10.97; *P* < 0.001). In addition, post-hoc *t*-tests with Bonferroni correction for multiple testing revealed that the LF:HF ratio varied significantly in the control group between the baseline and resting period (*P* < 0.001) and between the baseline and distention conditions (*P* = 0.002), but the LF:HF ratio was not significantly different between the baseline and resting period with the bag (*P* = 0.62). However, the LF:HF ratio was not significantly different between these three conditions in the non-constipated IBS group (*F*_(2, 47)_ = 0.29; *P* = 0.75).

#### Parasympathetic activity (HF)

After the barostat bag placement, the HF value increased compared with the baseline and decreased during colorectal distention compared with the resting period with the bag (main effect of condition, *F*_(2, 111)_ = 208.8; *P* < 0.0001). We observed no main effect of group (*F*_(2, 57)_ = 1.38; *P* = 0.25) or sex (*F*_(1, 57)_ = 0.3; *P* = 0.58), nor a group × condition interaction effect (*F*_(2, 111)_ = 2.14; *P* = 0.12; Fig. 1B).

### Association between autonomic activity and perception of colorectal distention

#### Baseline autonomic parameters and colorectal distention toleration threshold

**Figure 2:**
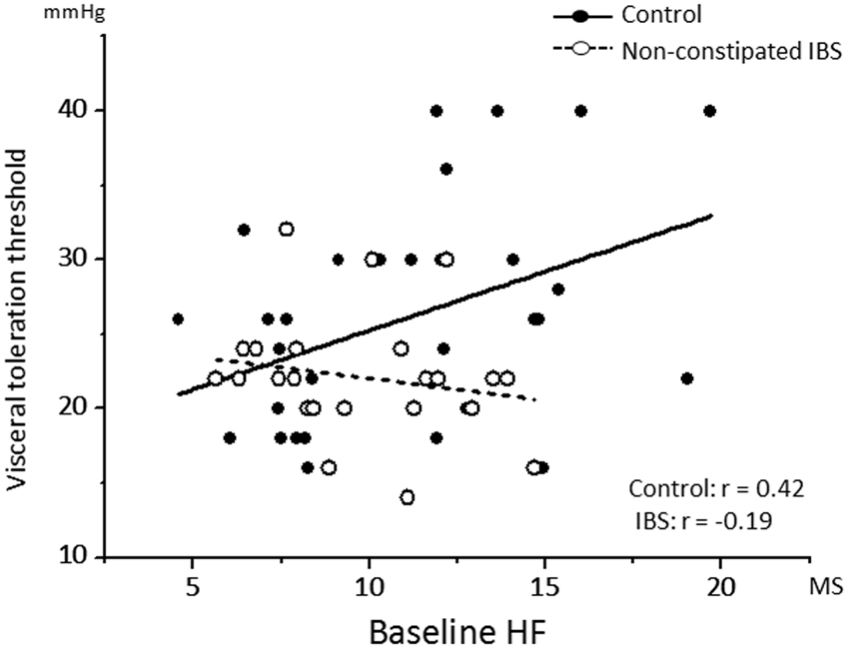
A scatter plot of the correlation between the baseline parasympathetic vagal tone (HF) and visceral perception threshold. Regression lines are shown per group. HF, high frequency; IBS, irritable bowel syndrome. MS, meter second.

A significant baseline HF value × group (controls and non-constipated IBS) interaction effect was observed (*F*_(1, 1)_ = 4.24; *P* = 0.04; Fig. 2). We separately performed a correlation analysis in each group to follow-up on this significant interaction effect. A significant correlation was established between the baseline HF value and colorectal distention toleration threshold in the control group (*r* = 0.42; *P*_(*Holm*)_ = 0.04), but not the non-constipated IBS group (*r* = −0.19; *P*_(*Holm*)_ = 0.36). A significant baseline LF:HF ratio × group interaction effect was also observed on colorectal distention toleration threshold (*F*_(1, 1)_ = 5.01; *P* = 0.03). The correlations in each group separately had an opposite sign, but were not significant (control group: *r* = − 0.28, *P*_(*Holm*)_ = 0.14 and non-constipated IBS groups: *r* = 0.36, *P*_(*Holm*)_ = 0.14).

#### Autonomic response to distention and colorectal distention toleration threshold

We calculated changes in values between the resting period with the bag and distention periods as ΔHF and Δ(LF:HF). We observed no significant ΔHF value × group interaction effect, nor was there a significant Δ(LF:HF) ratio × group interaction effect.

### Association between autonomic activity and brain activity during colorectal distention

The subjects participated in functional magnetic resonance imaging (fMRI) with colorectal distention on a different day from the autonomic activity measurement. The autonomic parameters calculated above [baseline HF and LF:HF and ΔHF and Δ(LF:HF)] were used as individual markers of autonomic activity. We performed an exploratory whole brain analysis.

#### Group difference in the association between autonomic activity and brain response

**Figure 3:**
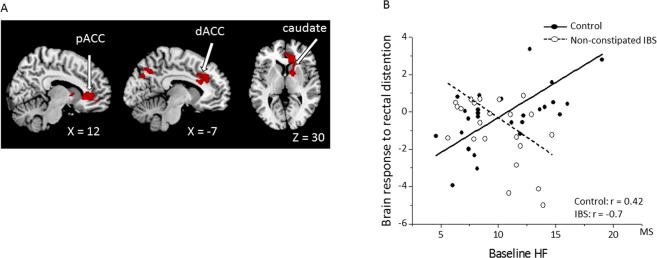
Brain activity during colorectal distention associated with the baseline parasympathetic vagal (HF) tone. (**A**) The activity in the dACC, pACC, and right caudate from the whole-brain analysis. Red, significant voxels (uncorrected *P* < 0.001 at the voxel level combined with a FWE-corrected *P* < 0.05 at the cluster level). (**B**) Scatter plot and regression of the brain activity during colorectal distention (vs. no distention) and baseline HF values in patients with non-constipated IBS compared to controls for the pACC. Abbreviations: dACC, dorsal anterior cingulate cortex; HF, high frequency; IBS, irritable bowel syndrome; MS, meter second, pACC pregenual anterior cingulate cortex.

We observed a significant difference in the correlation between the baseline HF value and the neural response to rectal distension between groups in the right pregenual anterior cingulate cortex (pACC), caudate, pallidum, superior frontal gyrus, putamen, middle frontal gyrus, and bilateral dorsolateral anterior cingulate cortex (dACC), midcingulate cortex (MCC), precuneus, and cuneus. A positive correlation between the baseline HF value and the brain response in the control group, which was absent in the non-constipated IBS group, resulted in a significant interaction effect in these regions (Fig. 3A; Table 4). Figure 3B presents the association between the brain activity during rectal distention (compared to no distention) and the baseline HF values in the pACC, which revealed a significant positive correlation in the control group (*r* = 0.42; *P* = 0.01) and a significant negative correlation in the IBS group (*r* = –0.7; *P* < 0.001). No significant difference was revealed in the correlation between the baseline LF:HF ratio, ΔHF value, or Δ(LF:HF) ratio and brain responses during rectal distention (versus no distention) between the control and non-constipated IBS groups.

**Table 4:** The correlation between baseline HF values and brain activity during rectal distention (versus no distention): Whole-brain analysis. In each cluster, all local maxima at least 10 mm apart are shown.

Side	Region	P_FWEcor_	Cluster	P_FWEcor_	T	X	Y	Z
		(cluster)	Size (voxels)	(peak)	(peak)	(mm)		
**Interaction**								
Control > non-constipated IBS								
R	pACC	0.002	1794	0.038	5.31	11	30	−5
R	Caudate/Pallidum			0.421	4.28	15	5	4
R	Caudate			0.924	3.60	18	17	6
R	Superior fontal gyrus			0.968	3.46	11	48	−6
R	Putamen			0.983	3.38	26	3	6
R	Middle frontal gyrus			0.985	3.36	26	53	7
LR	Precuneus	0.043	913	0.167	4.72	0	−70	43
R	Precuneus/Cuneus			0.572	4.10	−6	−75	33
L	dACC	0.019	1033	0.348	4.38	−5	33	33
L	dACC/MCC			0.386	4.33	−8	23	21
R	Superior frontal gyrus			0.901	3.65	5	39	24
LR	dACC/MCC			0.918	3.61	5	26	28
Control < IBS								
No significant findings								
**Control**								
Positive								
L	Precuneus	0.021	862	0.069	5.937	−6	−63	41
LR	Precuneus			0.156	5.463	3	−71	42
R	pACC	0.027	807	0.484	4.743	18	39	12
R	pACC/ Superior frontal gyrus			0.783	4.282	20	42	−3
R	Superior frontal gyrus			0.996	3.521	20	48	18
Negative								
No significant findings								
**IBS**								
Positive								
No significant findings								
Negative								
No significant findings								

Abbreviations: IBS, irritable bowel syndrome; dACC, dorsal anterior cingulate cortex; MCC, midcingulate cortex, pACC, pregenual anterior cingulate cortex.

#### Association between autonomic activity and brain response in each group

In the control group, the baseline HF value positively correlated with brain responses to rectal distention (versus no distention) in the bilateral precuneus, right pACC, and right superior frontal gyrus (Table 4). We observed no significant correlation of the baseline LF:HF ratio, ΔHF value, or ΔLF:HF ratio with brain responses in the control group. In the non-constipated IBS group, no significant correlation of the baseline HF value, LF:HF ratio, ΔHF value, or ΔLF:HF ratio with brain responses was observed during rectal distention.

## Discussion

This study examined the association between ANS function and the perceptual and brain response to colorectal distention in healthy subjects and non-constipated IBS patients. The association differed between HCs and patients with non-constipated IBS. First, patients with non-constipated IBS demonstrated a blunted sympathovagal balance (LF:HF ratio) response to colorectal distention compared with controls. Next, HCs with a higher baseline parasympathetic activity (HF component) had higher toleration thresholds for colorectal distention. In addition, those with a higher baseline parasympathetic activity exhibited enhanced brain responses to colorectal distension in visceral signal processing and modulation areas, including the right caudate, pACC, and dACC in whole-brain analysis. In contrast to HCs, patients with non-constipated IBS did not display any correlation between baseline parasympathetic activity and perceptual and brain responses to colorectal distention.

In response to visceral stimulation, cardiovascular autonomic changes normally increase sympathetic tone and facilitate parasympathetic vagal withdrawal^7^. HCs showed increased sympathovagal balance (LF:HF ratio). In contrast, the non-constipated IBS group demonstrated a blunted sympathovagal balance response. This may indicate ANS malfunction in patients with IBS, as reported repeatedly before^31–34^. Several previous studies measured ANS activity at rest and in response to visceral stimulation or visceral stressors^31–34^; however, the results were inconsistent. Some studies reported that IBS is linked to higher cardiosympathetic tone and/or lower cardiovagal tone compared to healthy controls^32,34^ while other studies revealed no difference in ANS measures between the IBS and control groups at baseline^31^. A recent study reported that patients with IBS had significantly diminished cardiosympathetic and cardiovagal (parasympathetic) responsiveness, both leading up to and following sigmoidoscopy^31^. In addition, patients with IBS exhibited increased parasympathetic activity and decreased sympathetic reactivity in response to cold water immersion of the forefoot, compared to the responses observed in controls^35^. Furthermore, blunting of ANS responses in patients with IBS is more pronounced as the disease progresses, suggestive of ANS “wear and tear” in these patients^31^. Patients with fibromyalgia exhibit lower sympathetic reactivity to cold pressor pain^23,36^. Likewise, our finding of a blunted sympathovagal balance response to colorectal distention supports hypothesized ANS blunting in patients with non-constipated IBS. Basically, the ANS plays a role in moment to moment control of peripheral function in adaptation to internal and external environmental change. The current result indicates that patients with IBS may exhibit blunted flexibility in their ANS reactions. However, considering the relatively small sample size of the current study and heterogeneity of IBS, we have to be cautious to generalize this result to all IBS patients.

In HCs, participants with higher baseline parasympathetic activity exhibited lower colorectal distention toleration thresholds, but this was not seen in patients with non-constipated IBS. There is a well-recognized relationship between parasympathetic activity and pain perception^24,37,38^. Underlying mechanisms likely include the pain-inhibiting role of the parasympathetic vagus nerve; vagal afferents end in the NTS, which provides descending pain inhibition to nociceptive transmission via other brainstem nuclei such as PBN and PAG^39^. While vagotomy augments pain, vagal stimulation reduces pain in both animals^40^ and humans^41,42^. This indicates that both vagal afferents and efferents may contribute to pain perception. Studies have reported correlations between resting parasympathetic activity and pain perception^20–23,37,43^. In addition, lower parasympathetic activity, measured as HRV, correlated with higher pain sensitivity to thermal stimuli in patients with fibromyalgia^23^, those with chemotherapy-induced neuropathy^24^ and controls^20–22^. Epidemiologically, lower resting vagal tone correlates with extended pain-related sick leave^44^. Our findings are consistent with these previous studies, in that we also identified that HCs with higher resting parasympathetic activity had higher pain tolerance. On the other hand, autonomic parameter responses to colorectal distention [ΔHF and Δ(LF:HF)] were not correlated with the toleration threshold. This may be due to the higher variability of HRV during the distention condition, while the resting condition may be able to more reliably capture individual differences in autonomic function. Notably, in contrast to HCs, patients with non-constipated IBS did not show a correlation between parasympathetic activity and colorectal distention toleration threshold. A previous study showed that parasympathetic HRV measures are significantly lower in females with IBS who report high abdominal pain^45^. In contrast, our findings indicate failure of the functional interaction between ANS and visceral sensitivity, rather than decreased ANS activity in patients with non-constipated IBS. Considering the nature of the broad ANS function, which maintains homeostasis of the body, it may be more relevant to study its role related to other functions such as visceral sensitivity rather than simply focus on increase or decrease of ANS activity, as we did in the present study. We may speculate that this is due to a shift of the autonomic activity under the long-term pathological condition in IBS, or vulnerability of autonomic function in IBS influenced by early life events^46^.

The most novel finding of this study was the correlation between the parasympathetic activity at rest and brain activity in the pACC, dACC, and right caudate during colorectal distention in the HC group. The pACC, dACC, caudate, and brainstem are activated during pain processing,^15,47^ and pain modulation, as well as related to the CAN^19^. Placebo administration reduces cortisol plasma levels, subjective pain, and μ-opioid system activation in the dACC and PAG^48^. In addition, the dACC is the target of pain modulation through real-time fMRI neuromodulation^49^ and deep brain stimulation^50^. The dACC is one of the primary targets of top-down modulation of pain experience, as well as a central region that regulates autonomic outputs. The dACC significantly correlates with autonomic regulation in task-evoked brain activity^51^ and resting blood flow^52^. In addition, the pACC is critically involved in the parasympathetic regulation through bidirectional connections with the dorsal vagal complex, amygdala, PAG, and hypothalamus^53^. Furthermore, a descending pain modulation circuit is known to arise in the pACC feeding to the PAG^10^. Activation of the caudate nucleus can either elicit or suppress pain^54,55^, and the caudate is involved in acupuncture analgesia through μ-opioid receptors^56^. While caudate activation correlated with the baseline parasympathetic component of the HRV during inhibitory control^57^, HF-HRV negatively correlated with the grey matter volume in the right caudate^58^. Thus, the caudate seemingly plays an essential role in sensory processing and suppression of pain^55^, as well as parasympathetic regulation^58^.

In HCs, the brain areas correlating with the baseline parasympathetic activity are related to visceral nociceptive processing and modulation. As participants with a higher baseline HF parasympathetic activity presented with higher visceral toleration thresholds, these brain areas may play a vital role in mediating the functional interaction between parasympathetic function and visceral perception. In contrast to HCs, patients with non-constipated IBS did not show brain areas that correlated significantly with autonomic parameters, including baseline parasympathetic activity. A previous meta-analysis demonstrated altered brain processing of rectal distention in areas such as the pACC, dACC, insula, and midbrain^47^, and deficits of descending corticolimbic inhibition in the dorsal brainstem^18^ were reported in patients with IBS. The current study suggests a deficient functional coupling between the ANS and visceral perception in the above-mentioned areas, in patients with non-constipated IBS, rather than simple changes in brain activity when comparing them to HCs. Although it has been difficult, this study tried to characterize the functional interaction between ANS and visceral perception, mediated by the related brain areas in HCs and patients with non-constipated IBS. Our results carry two possible implications. Firstly, the relations found in HCs may no longer hold under a pathological condition, such as IBS, because it is not necessarily an extreme case of a distribution found in HCs. Secondly, many studies interpret peripheral parameter changes, such as those of the ANS, on the assumption that brain circuits that regulate peripheral function, or peripheral factors that influence brain function, work similarly in HCs and patients. However, the circuit itself may have been changed in patients with pathological conditions such as IBS^59^. Conceptualized as a disorder of brain-gut communication, more complex, hierarchical changes may be creating the symptoms expressed in IBS. Changes in peripheral parameters are often inconsistent in patients with IBS. Rather than due to one common parameter abnormality, factors intertwine with each other to produce the same symptoms.

This study has some limitations. First, sex differences in autonomic reactivity have been reported in both HCs^37^ and patients with IBS^5^. Although we sex-matched both the non-constipated IBS and control samples and controlled for the primary effects of sex, the number of participants was not sufficient to reveal sex differences (aside from differences between HCs and patients with non-constipated IBS). Second, we did not control for menstrual cycle in female participants because all participants attended different three-day sessions consisting of physiological examination of autonomic function and visceral sensation to rectal distention, structural MRI, and fMRI during rectal distention. Thus, controlling for the menstrual cycle was not feasible because of limitations in available equipment. In addition, IBS subtypes were limited to IBS-D and IBS-M. Hence, the applicability our findings might be limited to these variants. We cannot determine the influence of the mixture of IBS-D and IBS-M because the sample size of the IBS-M group is too small. Finally, HRV is a proxy for ANS activity and we cannot estimate whole ANS function only from HRV. In addition, it is a debatable issue whether the LF:HF ratio is truly a maker of sympathovagal balance as recent literature suggests that LF power has a poor relationship to sympathetic nerve activation, as well as non-linear interactions between sympathetic and parasympathetic nerve activity^60^. We also did not assess respiration in autonomic function measurement; the putative impact of respiration on autonomic parameters was not controlled. We therefore need to be cautious, although the current paper used the LF: HF ratio based on the traditional cardio-autonomic guidelines^61^.

In conclusion, this study aimed to characterize the functional interaction between the ANS and visceral perception, as mediated by related brain areas in HCs and non-constipated IBS. HCs with a higher parasympathetic tone at baseline exhibited lower sensitivity to rectal distention and significantly higher activation in the pACC, dACC, and right caudate during rectal distention. This suggests an adaptive functional interaction between parasympathetic function and visceral perception in these brain areas in health. In contrast, these interactions were not observed in patients with non-constipated IBS, and these patients exhibited blunted LF:HF sympathovagal balance to colorectal distention, compared with HCs. This suggests that a deficiency in functional coupling between the ANS and visceral perception in the above-mentioned areas may play a role in the pathophysiology of non-constipated IBS. Coupling of brain-mediated ANS activity and visceral perception may be altered in patients with non-constipated IBS, suggesting that more complex, hierarchical changes may be creating IBS symptoms, considered a brain-gut disorder.

## Methods

### Subjects

All participants completed the following questionnaires: the Japanese version of IBS-SI^28^, SDS^26^, and STAI^27^. Of note, participants in this study partially overlap with our previously published studies; however, the research questions are entirely different from those previously investigated^62,63^. In our previous studies, the response to the corticotrophin releasing hormone or influence of the anticipation on brain processing of colorectal distention were assessed, while here we estimated the individual differences of autonomic activity on perception and brain response to colorectal distention.

### Ethics

This study was approved by the Ethics Committee of the Tohoku University School of Medicine (Sendai, Japan) and conducted in accordance with the Declaration of Helsinki guidelines. We obtained written informed consent from all participants after explaining them the study protocol.

### Study design for autonomic activity and perception of colorectal distention

First, the baseline ECG was measured for 5 min with the subject in a reclined supine position. Next, a 10 cm, 700 mL capacity polyethylene bag was inserted into the rectum. With AML, we assessed the toleration threshold in the colorectum using a computer-controlled barostat pump (Polygram for Windows SVS module; Synectics Medical). Phasic distention lasted 30 s and was separated by 30 s rest intervals, starting at 4 mmHg and increased in steps of 4 mmHg until either a subject requested the protocol to be stopped or a pressure of 40 mmHg was reached. The toleration threshold of each subject was determined for use in the subsequent tonic distention period.

After this step, we measured ECG for a 3 min resting period with the bag in place but not inflated and during a 3 min tonic distention period at the individually titrated toleration threshold of each subject to quantify the ANS responses to colorectal distention. Immediately after tonic distention, all subjects had to assess the intensities of pain, urgency, and discomfort by rating using a 10 point scale (0 = “no sensation” and 10 = “worst sensation ever”).

#### ECG measurements

The ECG was measured with Ag/AgCl electrodes applied on the lower left side of the thorax and on the right shoulder. The ECG was recorded with a sampling frequency of 500 Hz and analyzed using an electrophysiology analysis program (BIMUTAS II; Kissei Comtec, Matsumoto, Japan). Digitized ECG signals were analyzed using in-house developed programs written in MATLAB (Math Works, Natick, MA, USA). We corrected occasional erroneous detections of the RR-wave recognitions in the ECG with a semiautomatic procedure that used a parabolic interpolation to increase the accuracy of R-wave recognition and which allowed minimal input from the investigator. A time-frequency analysis based on a fast Fourier transform using the spectrogram function with a moving Hamming window of 37.5 s (128 samples) included in the Signal Processing Toolbox of MATLAB was performed on continuous 2 min segments (epochs) of data. R-R intervals were calculated, resampled at the rate of 3.4 Hz, and interpolated to yield HRV signals for (1) a 5 min baseline period before the insertion of the barostat bag, (2) a 3 min rest period before tonic distention, and (3) a 3 min tonic distention period at the toleration threshold level. We analyzed the segments using spectral analysis characterizing heart rate autoregulation by calculating endogenous cardiac activity cycles, such as vagus-mediated respiratory sinus arrhythmia. According to frequencies in the HRV spectral analysis, ANS influences were discerned. The percent power of the HF band of the HRV power spectrum can be used as a marker of vagal tone defined as the percent power in the 0.15–0.40 Hz range, and the LF band in the 0.04–0.15 Hz range reflects sympathetic activity but can include vagal influence. Therefore, the percent power in the HF band as a measure of vagal tone and the LF:HF ratio as an indicator of sympathovagal balance were calculated for each segment.

### Statistical analyses of subject characteristics, perception, and autonomic activity

We analyzed data using SAS version 9.4 software (SAS Institute, Cary, NC). Data were expressed as mean ± SEM unless otherwise stated. A two-tailed *P* < 0.05 was considered statistically significant. In addition, we performed the Shapiro–Wilk test to assess continuous variables for normality. In cases of non-normality, we used a natural logarithmic transformation to normalize the distribution. In case normality could not be achieved after this transformation, we used non-parametric statistical methods. Furthermore, we compared psychological questionnaires between groups using independent-sample Student’s *t*-test with the stepdown Bonferroni (Holm) correction for multiple comparisons.

We used linear mixed models to assess autonomic responses to colorectal distention. In linear mixed model analyses, we modelled data by either fitting subject-specific intercepts and linear and quadratic effects of time as a continuous variable (random effects model) or by stipulating the most suitable variance–covariance matrix for residuals (marginal model) using a random or repeated statement in the proc mixed SAS procedure, respectively. In the latter case, a dissimilar variance–covariance matrix was used for each level of group and sex based on the observed variance–covariance matrix, resulting in the better model fit, which was chosen based on the lowest value of Akaike’s information criterion. While condition (baseline, resting, or distention periods) was included as a within-subject categorical independent variable, both group (non-constipated IBS vs. control) and sex were included as between-subject independent variables. In addition to main effects, we added the condition × group interaction effect in the model. In the case of a significant condition × group interaction, within-group post-hoc analysis was performed using paired Student’s *t*-tests with stepdown Bonferroni correction for multiple comparisons. Besides, a general linear model was used to assess the between-group difference in association with the autonomic activity and visceral pain perception. Furthermore, we calculated changes in values between the pre-distention, rest, and distention periods as ΔHF and Δ(LF:HF). The visceral sensation (toleration threshold) × group interaction effect was involved in the model (accompanied by both main effects) for each autonomic parameter (baseline HF, baseline LF:HF, ΔHF, and Δ(LF:HF)). Finally, we performed Pearson’s correlation post hoc tests with stepdown Bonferroni (Holm) correction for multiple comparisons in each group in case of a significant between-group difference. As anxiety may influence the autonomic parameters^43^, the associations between trait anxiety score and the autonomic parameters were assessed by Pearson’s correlation tests. In addition, the influence of IBS severity on the autonomic parameters was tested only in patients with non-constipated IBS by Pearson’s correlation tests.

### Brain imaging acquisition and study design

#### fMRI data acquisition

All neuroimaging data were acquired using a 3T SIEMENS MAGNETOM TrioTim scanner with a 32-channel head-coil. Functional images were collected using an echo-planar imaging sequence with blood oxygen level-dependent (BOLD) contrast (TR/TE = 3000/30 ms, voxel size = 2.5 × 2.5 × 2.5 mm^3^, flip angle = 90°, 50 slices) covering the whole brain including the cerebellum. 240 images were acquired per functional run for a total examination duration of 1.2 h. A high-resolution structural MRI image was acquired using a three-dimensional T1-weighted magnetization prepared rapid acquisition gradient echo sequence (TR/TE = 2800/2.8 ms, voxel size = 1.0 × 1.0 × 1.1 mm^3^) on a different day than the fMRI scan.

#### Brain imaging experimental design

The fMRI experimental design was as follows: six runs of 12 trials each. Each trial comprised an anticipation visual cue (symbol presented for 3 s), a fixation point (until the end of the distention period, 24–33 s), a period of distention or no distention (18 s) and a rating period. Three different anticipation cues (“!”, “0”, and “?”) indicated the rate of occurrence of the following distention (100%, 0%, and uncertain (50%), respectively). The previous study provides further details on the design and data acquisition^62,63^. In this study, we focused on brain processing of colorectal distention. The colorectal distention was induced using a barostat system (G&J Electronics Inc., Toronto, Canada) and stimulation level of the each subject was determined based on the discomfort threshold (the bag volume eliciting 40–60% discomfort).

### Statistical analysis of brain imaging data

We used data of 22 patients with non-constipated IBS and 26 controls for the final analysis; others were excluded because of excessive head movement during the fMRI scan.

#### Pre-processing

fMRI data were pre-processed and analysed using SPM8 (Wellcome Trust Centre for Neuroimaging, UCL). We performed spatial realignment to correct for small movements, slice timing, co-registration of the functional and structural images, segmentation of the structural image, and warping (including a 12-parameter affine transformation and a high-dimensional non-linear warping field of 120 × 145 × 121 × 3 parameters) to the Montreal Neurological Institute (MNI) space based on the structural image and the transformation obtained during the segmentation step. Furthermore, all warping parameters were applied to the functional images, which were then smoothed with a Gaussian isotropic 3D kernel with a full width at half maximum of 8 mm.

#### First (individual) level

We used a combined event (anticipation cue) and block (distention/non-distention period) design to statistically analyze the data using a generalized linear model in SPM8. In this study, each condition was modelled as a box-car stimulus function (block conditions) or stick function (event-related conditions), convolved with the canonical hemodynamic response function, and used in a standard generalized linear model that included high-pass filtering with a cut-off frequency of 1/128 s to remove LF drifts in signals. For each run, eight regressors of interest corresponding to the three anticipation types (i.e., 100% (“!”), 0% (“0”) and uncertain (“?”)), four distention conditions (i.e., distention-after “!”, distention-after “?”, non-distention-after “?”, and non-distention-after “0”), and a “rest” condition were defined. Of note, the impact of variable anticipation contexts on the brain activity during colorectal distention in non-constipated IBS has been reported previously^62,63^. Furthermore, in this study we computed only the contrast between distention following the 100% anticipation cue versus non-distention after the 0% anticipation cue for each subject to test the correlation between brain activity during distention and autonomic response.

#### Second (group) level

We conducted a whole-brain voxel-based analysis using SPM8 at a voxel-level threshold of *P*_uncorrected_ < 0.001 combined with a cluster-level threshold of *P*_FWE-corrected_ < 0.05. We used contrast images generated in the first-level analysis, which were the distention effect (distention following the 100% anticipation cue vs. non-distention after the 0% anticipation cue) to assess random effects at the group level. In addition, regression analyses in SPM8 were performed to evaluate correlations between brain responses and individual autonomic parameters for each group, followed by comparing these correlations between both groups, controlling for sex and age as nuisance variables. Furthermore, as a measure of brain activity, the first eigenvariate in the significant cluster from the between-group regression analyses was used to visualize the difference in association between both groups.

## Data Availability

The datasets generated during and/or analyzed during the current study are available from the corresponding author on request
